# PRISM: privacy-preserving rare disease analysis using fully homomorphic encryption

**DOI:** 10.1093/bioinformatics/btaf468

**Published:** 2025-08-23

**Authors:** Güliz Akkaya, Nesli Erdoğmuş, Mete Akgün

**Affiliations:** Department of Computer Engineering, İzmir Institute of Technology, Izmir, 35430, Turkey; Department of Computer Engineering, İzmir Institute of Technology, Izmir, 35430, Turkey; Department of Computer Science, University of Tübingen, Tübingen, 72076, Germany; Institute for Bioinformatics and Medical Informatics, University of Tübingen, Tübingen, 72076, Germany

## Abstract

**Motivation:**

Rare diseases affect millions of people worldwide, yet their genomic foundations remain poorly understood due to limited patient data and strict privacy regulations, such as the General Data Protection Regulation (GDPR) (https://gdpr.eu/tag/gdpr/) in March 2025. These restrictions can hinder the collaborative analysis of genomic data necessary for uncovering disease-causing variants.

**Results:**

We present PRISM, a novel privacy-preserving framework based on fully homomorphic encryption (FHE) that facilitates rare disease variant analysis across multiple institutions without exposing sensitive genomic information. To address the challenges of centralized trust, PRISM is built upon a Threshold FHE scheme. This approach decentralizes key management across participating institutions and ensures no single entity can unilaterally decrypt sensitive data. Our method filters disease-causing variants under recessive, dominant, and *de novo* inheritance models entirely on encrypted data. We propose two algorithmic variants: a multiplication-intensive (MUL-IN) approach and an addition-intensive (ADD-IN) approach. The ADD-IN algorithms minimize the number of costly multiplication operations, enabling up to a 17× improvement in runtime for recessive/dominant filtering and 22× for *de novo* filtering, compared to MUL-IN methods. While ADD-IN produces larger ciphertexts, efficient parallelization via SIMD and multithreading allows it to handle millions of variants in reasonable time. To the best of our knowledge, this is the first study that utilizes FHE for privacy-preserving rare disease analysis across multiple inheritance models, demonstrating its practicality and scalability in a single-cloud setting.

**Availability and implementation:**

The source code and the data used in this work can be found in https://github.com/mdppml/PRISM.git.

## 1 Introduction

Rare diseases affect millions globally (https://rarediseases.org/rare-diseases/), significantly impairing quality of life [e.g. cystic fibrosis complications (https://www.nhlbi.nih.gov/health/cystic-fibrosis/symptoms), pediatric cancers]. Advancing diagnosis and treatment relies heavily on genomic data, but accessibility is limited due to patient data scarcity per institution and strict privacy regulations. Collaborative analysis across institutions is essential but hindered by these restrictions. Secure multi-party computation (MPC) (Jagadeesh *et al.* 2017, Akgün et al. 2021) offers solutions but often requires multiple non-colluding servers, a potentially challenging assumption, and can incur significant communication overhead (Yao 1986).

To overcome these limitations, this study introduces a privacy-preserving collaborative method for protecting patients’ genome data through the use of fully homomorphic encryption (FHE) on a single-cloud platform. FHE allows for an arbitrary number of computations on encrypted data without requiring decryption, thereby ensuring data confidentiality throughout the entire process. The proposed method facilitates rare disease research by enabling computations on encrypted genome data collected from various medical institutions while maintaining privacy. The encrypted genome data of rare disease patients are processed using arithmetic operations, namely addition and multiplication, to identify disease-causing variants within a cloud environment. This analysis is conducted considering three specific inheritance models: recessive, dominant, and *de novo*. In this work, we make the following key contributions:

- Decentralized privacy-preserving framework. We develop a framework based on Threshold FHE that enables secure collaboration among multiple institutions. By distributing the secret key into shares held by a Key Management Committee, our model removes the single point of trust and cryptographically ensures that no single party can access the sensitive data, directly addressing the limitations of a single-key setup.
- Inheritance model support. We design specialized algorithms for three inheritance models—recessive, dominant, and *de novo*—enabling a comprehensive range of analyses for rare disease genomics. Two algorithmic variants (MUL-IN and ADD-IN) are introduced, balancing execution speed and ciphertext size.
- Practical scalability. Our framework leverages single instruction multiple data (SIMD) capabilities and multithreading to process millions of variants efficiently. Experimental results show that reducing multiplication operations yields up to a 17× speedup for recessive/dominant filtering and a 22× speedup for *de novo* filtering, demonstrating real-world feasibility for privacy-preserving rare disease analysis at scale.

To our knowledge, this is the first study demonstrating practical, scalable FHE-based rare disease analysis across multiple inheritance models in a single-cloud setting.

The article is organized as follows: Section 2 reviews related work. Section 3 covers rare disease analysis and FHE concepts. Section 4 details the PRISM methodology. Section 5 analyzes security aspects. Section 6 presents experiments and results. Section 7 concludes.

## 2 Related work

Analyzing sensitive genomic data while preserving privacy necessitates privacy-enhancing technologies (PETs). Our work, PRISM, employs FHE for collaborative rare disease variant filtering based on inheritance models. This section contextualizes PRISM by reviewing relevant PET applications in genomics.

### 2.1 HE approaches

Homomorphic encryption (HE) enables computation on encrypted data. Early applications (Lauter *et al.* 2015) used partially or somewhat HE for basic GWAS statistics, often limited by performance. Subsequent work leveraged FHE for more complex tasks: HEALER (Wang et al. 2016) focused on secure logistic regression for rare variants, while FORESEE (Zhang *et al.* 2015b) targeted secure chi-square statistics for GWAS. Kim *et al.* (2021) used optimized HE (CKKS scheme) for genotype imputation. These works primarily address statistical analysis or imputation. In contrast, PRISM tackles the distinct computational logic of filtering variants based on Mendelian inheritance patterns (recessive, dominant, *de novo*) using the BFV scheme for exact integer arithmetic, essential for precise logical comparisons.

Multi-party homomorphic encryption (MHE) (Froelicher *et al.* 2021) allows multiple parties to compute jointly without a single trusted key manager, unlike PRISM’s single-key model. However, MHE generally incurs higher overhead. PRISM focuses on the feasibility and optimization (e.g. MUL-IN versus ADD-IN) of inheritance filtering within a practical single-key FHE framework. Other HE applications like secure relative discovery (Hong *et al.* 2024) also address different computational problems than PRISM’s variant filtering.

### 2.2 Secure MPC

MPC allows joint computation without revealing inputs. Implementations exist for GWAS (Zhang *et al.* 2015a), simple genomic diagnoses (Jagadeesh *et al.* 2017), and identifying disease mutations under inheritance models (Akgün *et al.* 2021). While powerful, MPC often requires significant communication rounds and bandwidth, potentially leading to latency, and relies on assumptions about non-colluding parties. PRISM’s FHE approach shifts the main bottleneck from communication to computation on a central server, eliminating inter-party interaction during computation. We experimentally compare PRISM’s performance against a state-of-the-art 2PC approach (Akgün *et al.* 2021).

### 2.3 Other PETs

Other PETs include trusted execution environments (TEEs) like Intel SGX (Chen et al. 2017), which rely on hardware security but require trust in the manufacturer and face potential side-channel vulnerabilities. Differential privacy (DP) (Wang *et al.* 2017) adds noise to provide formal privacy guarantees, but the inherent privacy-utility tradeoff can hinder the detection of rare variants. Federated learning (FL) (Sav *et al.* 2022) focuses on distributed model training rather than executing specific filtering queries like PRISM.

### 2.4 Positioning PRISM

While various privacy-preserving techniques have been applied to genomics (e.g. GWAS (Lauter *et al.* 2015, Froelicher *et al.* 2021), imputation (Kim *et al.* 2021)), work specifically addressing inheritance model-based filtering for rare diseases is less common. A notable example using MPC is Akgün *et al.* (2021), providing a key benchmark for our FHE-based approach. PRISM specifically utilizes FHE (BFV scheme) for collaborative rare disease variant filtering based on inheritance logic, a distinct task from most prior HE work focused on statistics or imputation. It provides exact results, crucial for filtering, unlike DP. Compared to MPC, PRISM offers a communication-light alternative, trading computational cost for reduced network interaction and different trust assumptions (relying on FHE’s cryptographic security). Unlike TEEs, it relies on cryptographic rather than hardware assumptions. PRISM demonstrates the practical feasibility and optimization potential of FHE for this critical genomic analysis task.

## 3 Preliminaries

This section represents fundamental concepts related to rare disease analysis and the FHE method.

### 3.1 Rare disease analysis

Researchers analyse rare diseases by examining patients’ genome data. They use genome sequencing as a research tool to identify variants within the genomes. Because each individual’s genome contains millions of variants, researchers must identify the disease-causing variants within this vast dataset to understand the underlying causes of rare diseases. The analysis is conducted using variant call format (VCF) files, which store the genome sequencing data of patients. These files are analysed by filtering variants according to specific inheritance models, such as recessive and dominant inheritance patterns, and by incorporating family information. This filtering process helps identify variants of interest, allowing researchers to focus their efforts on these particular variants to diagnose and develop treatments for rare diseases.

Inheritance models: As mentioned above, rare disease analysis focuses on prioritizing disease-causing variants in the entire genome data by considering specific inheritance models. The use of inheritance models is crucial for rare disease analysis, as disease-causing variants are inherited from parents. The inheritance models examined for privacy-preserving rare disease analysis in this study are described below.

- Recessive inheritance model: The affected individual inherits the disease-causing variant in a homozygous state, while both parents are heterozygous carriers of the variant.
- Dominant inheritance model: The affected individual is heterozygous for the disease-causing variant, having a single copy of the variant that is inherited from one parent.
- *De novo* inheritance model: All affected individuals possess the same type of disease-causing mutation, and this mutation is not present in any other unaffected family members.

**Figure 1.**
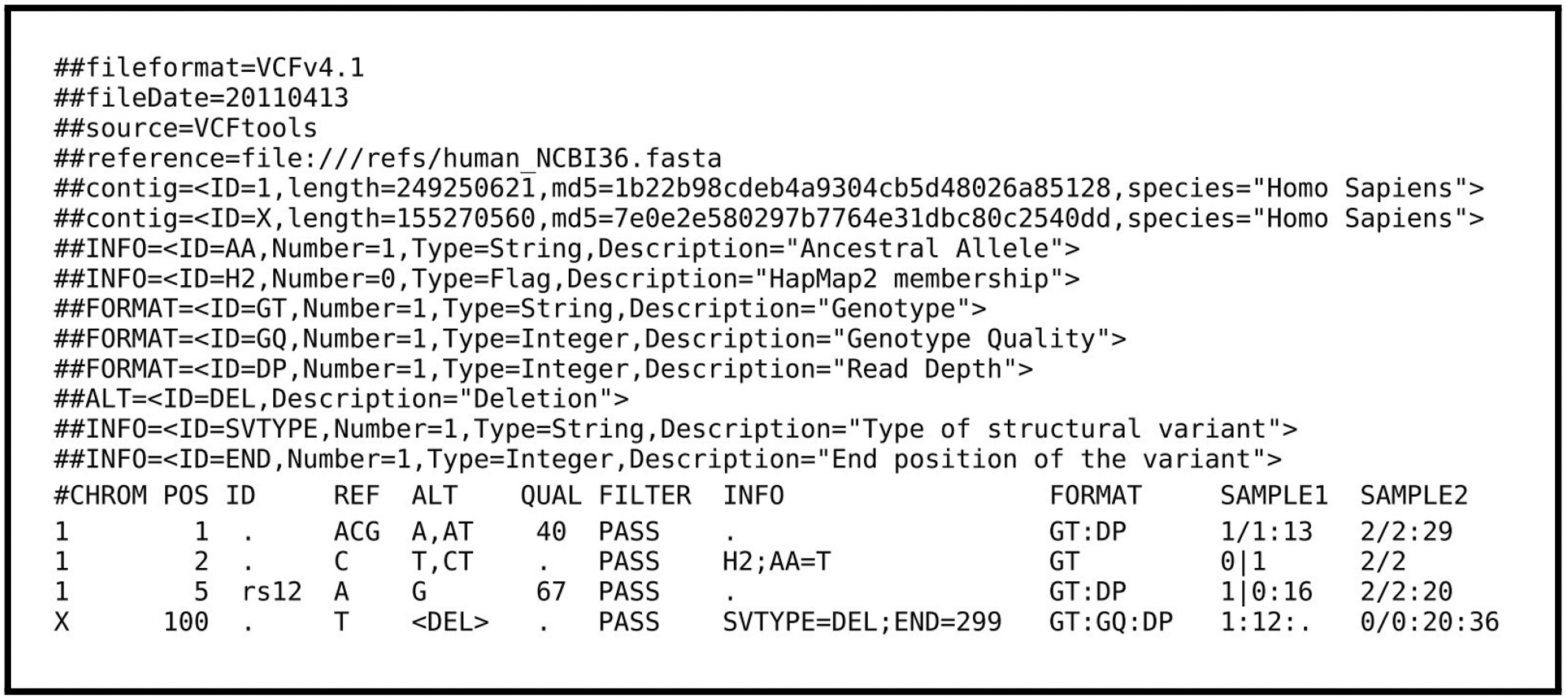
Sample VCF file (Danecek *et al.* 2011).

VCF: VCF (Danecek *et al.* 2011) is a standard format for representing and storing human genome sequence variation data. The genome variation data of rare disease patients are stored in VCF files, which researchers use to analyse disease-causing variants. A VCF file consists of two sections: the header and the data section. The header section contains descriptions of the tags and annotations used in the data section, including a field definition line that lists the names of the data section columns, such as chromosome, position, identifier, reference allele, alternate non-reference alleles, format, and sample IDs. The data section presents the data corresponding to the columns listed in the header. In the sample data column, variant data of patients appear as homozygous or heterozygous using numerical codes. An example of VCF file is shown in Fig. 1.

### 3.2 Homomorphic encryption

HE is a cryptographic method that enables computations to be performed on encrypted data without needing the decryption key. Rivest *et al.* (1978) introduced the term “homomorphism” for the first time in the field of cryptography to describe the technique that allows addition and multiplication operations on encrypted data without decrypting it.

FHE: FHE supports an unlimited number of addition and multiplication operations on encrypted data. Each addition or multiplication operation increases the noise level within the ciphertext. The multiplication operation, due to its greater computational complexity, contributes to this noise increase more significantly than addition. Once the noise level exceeds a certain threshold, the decryption function fails to recover the original message accurately. Therefore, it is crucial to reduce the noise level to ensure successful decryption of the ciphertext. The first practical example of an FHE scheme was introduced by Gentry (2009). This scheme is based on ideal lattices and incorporates two key techniques: squashing and bootstrapping. The squashing method reduces the complexity of decryption, while the bootstrapping method regenerates a ciphertext with a lower noise level from a noisy ciphertext. This reduction in noise allows for additional computations to be performed on the resulting ciphertext. Another example is the Brakerski–Fan-Vercauteren (BFV) scheme which enables modular arithmetic operations on encrypted integer data (Brakerski 2012, Fan and Vercauteren 2012). The BFV scheme is categorized as a leveled FHE scheme, with its security grounded in the ring learning with errors (RLWE) problem. Additionally, Bajard *et al.* (2017) introduced an optimization of the BFV scheme that utilizes the residue number system (RNS).

## 4 Methods

Accessing sufficient genome data for rare disease research is challenging due to privacy concerns and data fragmentation. PRISM employs FHE for privacy-preserving collaborative analysis, enabling secure access to pooled data.

**Figure 2.**
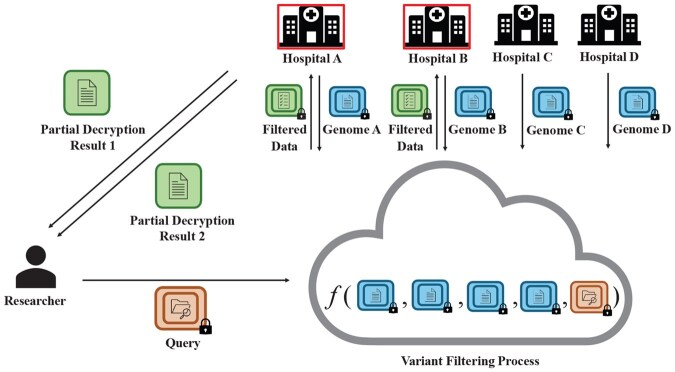
Privacy-preserving collaborative rare disease analysis scenario.

Multiple hospitals encrypt their numerically represented patient genome data. These encrypted data are pooled in a cloud environment. A researcher submits an encrypted query specifying filtering criteria based on an inheritance model. The cloud uses FHE to filter variants on the encrypted data. The filtered data are transmitted to the hospitals that are part of the key management committee. An interactive decryption protocol is then collaboratively executed by the committee members. Each member returns a partial decryption result to the researcher, who reconstructs the final filtered data by combining these partial results. The hospitals that are members of the committee are shown in the box in the figure (see Fig. 2).

The steps of the proposed method are detailed in the following sections, covering genome data representation, encryption of genome data, and inheritance model-based variant filtering, respectively.

### 4.1 Genome representation

The genome data, which includes the sample variant information of various individuals, is stored in VCF files. Within these files, variants are categorized as either homozygous or heterozygous. As the first step, it is necessary to represent the variant data numerically to employ the FHE method. The variant data stored in VCF files are encoded as integers using binary representations (0s and 1s) to enable compatibility with the FHE method. Disease-causing heterozygous variant is represented as 01, disease-causing homozygous variant is represented as 10, and homozygous reference (non-variant) is represented as 00. Examples of the numerical representations are shown in Table 1. In the table, x represents an integer number greater than 0.

**Table 1.** The variant data and descriptions.

Variant	Representation	Description
./.	00	Homozygous reference (non-variant)
0/0	00	Homozygous reference (non-variant)
x/0	01	Disease-causing heterozygous variant
x/x	10	Disease-causing homozygous variant

### 4.2 Encryption of genome data

The integers representing the variants of patients are encrypted using the BFV scheme. Due to the large number of variants, the data are partitioned into smaller blocks for encryption. Elements of each block are stored in a vector that is then encoded as plaintext. Next, each plaintext is encrypted with the 128-bit security level and stored as ciphertext in a file. During the encryption phase, different values of multiplicative depth are used depending on the algorithm. Multiplicative depth is a parameter that determines the maximum number of operations that can be performed on ciphertexts before the noise level exceeds the acceptable threshold.

### 4.3 Inheritance model based variant filtering

Privacy-preserving rare disease analysis is performed on the encrypted variant data using arithmetic operations such as addition and multiplication. This analysis considers recessive, dominant, and *de novo* inheritance models. Since the genome data is organized into blocks stored as vectors, SIMD enables a single arithmetic operation to be applied simultaneously to multiple elements within these vectors. Furthermore, multithreading is employed to process the blocks of genome data in parallel, significantly reducing computation time. The specific operations performed for each inheritance model are detailed below.

Recessive and dominant inheritance models: The privacy-preserving rare disease analysis is performed by filtering variants that match the query for recessive and dominant inheritance models. The filtering process employs arithmetic operations to compare encrypted variants with an encrypted query provided by the researcher, based on the selected inheritance model.

To compare the query with the patient variants, we propose a novel algorithm, which we refer to as the multiplication-intensive (MUL-IN) method, named for its reliance on a higher number of multiplication operations compared to our other proposed method. For each variant (each row in the VCF file), the comparison process is carried out across all samples in that row. Specifically, for each sample, the sample’s variant value is subtracted from the query value, and then the query value is subtracted from the sample’s variant value. The results of these two subtractions are multiplied, and the encrypted value of 1 is added to this multiplication result, yielding a comparison outcome for the sample. After this procedure is performed for all samples in the row, the individual comparison outcomes are aggregated by multiplication to form a single encrypted result that indicates whether the variant satisfies the query across the relevant samples. An encrypted value of 1 denotes that the variant passes the filter, while 0 signifies it does not. Finally, these results are summed in encrypted form to calculate the total number of variants matching the query, and this encrypted total is returned to the researcher. The steps of this algorithm are shown in Algorithm 1.

Algorithm 1. Variant filtering (MUL-IN method for recessive and dominant inheritance models)

**Require:** *V*[*i*]: A vector containing ciphertexts of all variants for sample *i*. *Q*[*i*]: A vector containing the ciphertexts of the *i*th query item, repeated for the total number of variants. *s*: The number of samples.

**Ensure:** *S* is a ciphertext representing the encrypted count of variants that pass the filter.

1: **for**  i←1 to *s* **do**

2:  $S_{1}[i]\leftarrow \mathrm{SUB}(V[i],Q[i])$  ▹ Subtract query value from variant value for sample *i*

3:  $S_{2}[i]\leftarrow \mathrm{SUB}(Q[i],V[i])$  ▹ Subtract variant value from query value for sample *i*

4:  $M[i]\leftarrow \mathrm{MUL}(S_{1}[i],S_{2}[i])$  ▹ Multiply subtraction results for sample *i*

5:  $R_{s}[i]\leftarrow \mathrm{ADD}(M[i],E(1))$  ▹ Add encrypted 1 to the multiplication result for sample *i*

6: **end for**

7: $R_{\text{alleles}}\leftarrow \mathrm{MULMANY}(R_{s})$  ▹ Perform cumulative multiplication over all samples in $R_{s}$

8: $R\leftarrow \mathrm{MUL}(R_{\text{alleles},\;\text{first}},R_{\text{alleles},\;\text{second}})$  ▹ Multiply results of first and second allelic values

9: S←SUM(R)  ▹ Sum filtering results to get the count of matching variants

An alternative algorithm is then employed to compare the variants in the genome data with the query. This algorithm, called as the addition-intensive (ADD-IN) method, aims to reduce computation time by minimizing the number of multiplication operations. Although the initial comparison steps for each variant sample remain the same as before, the ADD-IN approach relies more heavily on addition operations than on multiplication, thereby requiring fewer multiplications than the earlier method. Once the comparison results for all samples are obtained, their sum is computed to create a ciphertext representing the comparison results of each variant. The encrypted value corresponding to the total number of samples is then subtracted from this ciphertext. As a result, each variant’s comparison outcome is represented by an encrypted value of 0 if it passes the query or by another encrypted value within the range of (−1, 1 − *s*) in other cases. Furthermore, random encrypted values are multiplied with these results, followed by a shuffle operation, to ensure privacy by preventing the disclosure of information. Consequently, the final comparison result contains encrypted 0 values for the filtered variants or various other random encrypted values for the remaining variants. The steps of this process are given in Algorithm 2.

Algorithm 2. Variant filtering (ADD-IN method for recessive and dominant inheritance models)

**Require:** *V*[*i*]: A vector containing ciphertexts of all variants for sample *i*. *Q*[*i*]: A vector containing ciphertexts of the *i*th query item, repeated for the total number of variants. *s*: The number of samples. E(s): A ciphertext representing the encrypted count of samples. E(r): A ciphertext containing random values for privacy preservation.

**Ensure:** *R*: A ciphertext containing the shuffled filtering results for all variants.

1: **for**  i←1 to *s* **do**

2:  $S_{1}[i]\leftarrow \mathrm{SUB}(V[i],Q[i])$  ▹ Subtract query value from variant value for sample *i*

3:  $S_{2}[i]\leftarrow \mathrm{SUB}(Q[i],V[i])$  ▹ Subtract variant value from query value for sample *i*

4:  $M[i]\leftarrow \mathrm{MUL}(S_{1}[i],S_{2}[i])$  ▹ Multiply subtraction results for sample *i*

5:  $R_{s}[i]\leftarrow \mathrm{ADD}(M[i],E(1))$  ▹ Add encrypted 1 to the multiplication result for sample *i*

6: **end for**

7: $R_{\text{alleles}}\leftarrow \mathrm{ADDMANY}(R_{s})$  ▹ Perform cumulative addition over all samples in $R_{s}$

8: $R\leftarrow \mathrm{ADD}(R_{\text{alleles},\;\text{first}},R_{\text{alleles},\;\text{second}})$  ▹ Combine results for the first and second allelic values

9: $R\leftarrow \mathrm{SUB}(R,E_{s})$  ▹ Subtract encrypted sample count from the addition result

10: $R\leftarrow \mathrm{MUL}(R,E_{r})$  ▹ Multiply subtraction result with encrypted random values for privacy

11: **shuffle**(*R*) ▹ Shuffle the filtering results to ensure privacy

After the variant filtering process, the encrypted filtering results are sent to the researcher. Upon decrypting these results, the number of filtered variants is determined by counting the number of 0 values. This algorithm is designed to reduce computation time by minimizing the number of multiplication operations. However, it produces a larger ciphertext size compared to Algorithm 1, which is sent to the researcher.

*De novo* inheritance model: We propose two filtering processes that use arithmetic operations to identify which variants conform to the de novo criteria. First, we focus on a MUL-IN algorithm (Algorithm 3), which relies more heavily on multiplication than on addition operations. For each variant (i.e. each row in the data), the algorithm computes the product of all variant values for the affected samples, representing the presence of the variant in every affected individual. Then, it calculates 1−variant_value for each unaffected sample and multiplies these terms together, ensuring the absence of the variant in all unaffected individuals. Finally, these two products (for the affected and unaffected samples) are combined to yield a single encrypted result of either 0 or 1. A result of 1 indicates that the variant is present in all affected samples and absent in all unaffected samples, thus meeting the *de novo* condition, whereas a result of 0 indicates that it does not. This encrypted value is then transmitted to the researcher as the final result.

Algorithm 3. Variant filtering (MUL-IN method for the *de novo* inheritance model)

**Require:**  $V_{a}[i]$ is a vector containing ciphertexts of all variants for affected sample *i*. $V_{u}[i]$: A vector containing ciphertexts of all variants for unaffected sample *i*. *a*: The number of affected samples. *u*: The number of unaffected samples. E(1): A ciphertext representing the encrypted ones.

**Ensure:** *S*: A ciphertext representing the encrypted count of variants that pass the filter.

1: $M_{a}\leftarrow \mathrm{MULMANY}(V_{a})$  ▹ Cumulative multiplication of all affected samples’ variants

2: **for**  i←1 to *u* **do**

3:  $V_{u}^{\prime }[i]\leftarrow \mathrm{SUB}(E(1),V_{u}[i])$  ▹ Subtract each unaffected sample variant from encrypted value 1

4: **end for**

5: $M_{u}\leftarrow \mathrm{MULMANY}(V_{u}^{\prime })$  ▹ Cumulative multiplication of results for all unaffected samples

6: $R_{\text{alleles}}\leftarrow \mathrm{MUL}(M_{a},M_{u})$  ▹ Multiply the results of affected and unaffected samples

7: $R\leftarrow \mathrm{ADD}(R_{\text{alleles},\text{first}},R_{\text{alleles},\;\text{second}})$  ▹ Combine results for the first and second allelic values

8: S←SUM(R)  ▹ Sum the filtering results to compute the count of matching variants

The second algorithm aims to reduce computation time by limiting the number of multiplication operations and is termed the ADD-IN method because it uses more additions than multiplications. First, it sums all variant values for the affected samples, reflecting the presence of the variant in affected individuals. For each unaffected sample, it computes (1−variant_value) and sums these subtraction results, capturing the absence of the variant in unaffected individuals. The algorithm then combines these sums into a single ciphertext representing the filtering result for each variant and subtracts the encrypted number of samples from this ciphertext. Finally, to preserve privacy, the resulting ciphertext is multiplied by randomly chosen encrypted values and subjected to a shuffle operation, producing outcomes that are either encrypted zeros or other randomized ciphertexts. These steps, detailed in Algorithm 4, ensure that no sensitive information about individual sample values can be inferred from the final results.

Algorithm 4. Variant filtering (ADD-IN method for the *de novo* inheritance model)

**Require:**  $V_{a}[i]$: A vector containing ciphertexts of all variants for affected sample *i*. $V_{u}[i]$: A vector containing ciphertexts of all variants for unaffected sample *i*. *a*: The number of affected samples. *u*: The number of unaffected samples. E(a+u): A ciphertext representing the encrypted sum of affected and unaffected sample counts. E(r): A ciphertext containing random values for privacy preservation. E(1): A ciphertext representing the encrypted ones.

**Ensure:** *R*: A ciphertext containing the shuffled filtering results for all variants.

1: $A_{a}\leftarrow \mathrm{ADDMANY}(V_{a})$  ▹ Cumulative addition of variants for all affected samples

2: **for**  i←1 to *u* **do**

3:  $V_{u}^{\prime }[i]\leftarrow \mathrm{SUB}(E(1),V_{u}[i])$  ▹ Subtract each unaffected sample variant from the encrypted value 1

4: **end for**

5: $A_{u}\leftarrow \mathrm{ADDMANY}(V_{u}^{\prime })$  ▹ Cumulative addition of results for all unaffected samples

6: $A\leftarrow \mathrm{ADD}(A_{a},A_{u})$  ▹ Combine the addition results for affected and unaffected samples

7: $R_{\text{alleles}}\leftarrow \mathrm{SUB}(A,E(a+u))$  ▹ Subtract the encrypted total sample count from the addition result

8: $R\leftarrow \mathrm{MUL}(R_{\text{alleles},\;\text{first}},R_{\text{alleles},\;\text{second}})$  ▹ Multiply the results for the first and second allelic values

9: R←MUL(R,E(r))  ▹ Multiply the subtraction result with random values for privacy

10: **shuffle**(*R*) ▹ Shuffle the filtering results to ensure privacy

The encrypted results are transmitted to the researcher, who determines the number of filtered variants by counting the occurrences of 0 during decryption. While this algorithm reduces computation time by requiring fewer multiplications, it entails sending a larger ciphertext compared to Algorithm 3.

## 5 Security analysis

The privacy guarantees of the PRISM framework are founded on the semantic security of the underlying threshold homomorphic encryption scheme and our defined decentralized trust model.

### 5.1 System model and decentralized trust assumptions

The system involves three main parties: Data Owners (e.g. hospitals), a Cloud Server for computation, and a Researcher. To eliminate single points of failure, PRISM’s security is built upon a decentralized trust model.

Our trust model is defined as follows:

- Key Management Committee: A designated subset of consortium institutions (e.g. 3–5 members) forms a committee responsible for all key management operations.
- Decentralized trust: The core security assumption is that no more than a pre-defined threshold (t−1) of committee members will collude. The system’s security relies on this robust *t*-out-of-*n* cryptographic trust model rather than on organizational policies alone.
- Cloud server: The cloud is considered “honest-but-curious”; it will execute computations correctly but may try to infer information from the ciphertexts it processes. The semantic security of the BFV (Fan and Vercauteren 2012, Bajard *et al.* 2017) scheme prevents this.
- Researcher: The researcher is trusted to handle the final decrypted results responsibly but cannot bypass the committee’s authority.

### 5.2 Threshold key generation and management

PRISM’s key management protocol is designed to be fully decentralized among the committee members. The process is managed by the Key Management Committee and follows two main phases:

- Distributed key generation (DKG): The committee members engage in an interactive protocol using the OpenFHE (Badawi *et al.* 2022) library to collectively generate the system’s keys. The outcome is a single collective public key (*pk*), which is then distributed to all data owners for encryption. The corresponding secret key is never unified; instead, each committee member holds a unique secret key share ($sk_{i}$). This process cryptographically ensures that no single institution ever has access to the complete secret key.
- Interactive threshold decryption: After the researcher receives the final encrypted result from the cloud, she must initiate an interactive decryption protocol with the committee. The researcher sends the ciphertext to at least *t* members of the committee. Each member uses their secret share $sk_{i}$ to generate a partial decryption, which is sent to the researcher. The researcher then combines these partial decryptions to reconstruct the final plaintext result. This enforces the rule that a quorum of committee members must authorize access to the results.

### 5.3 Data and query privacy in PRISM outputs

The privacy of the final results returned to the researcher depends on the algorithm chosen, both of which protect the underlying data throughout the computation on the cloud.

- MUL-IN: This method returns an encrypted count of the matching variants. Upon decryption, the researcher learns only the total number of variants that passed the filter, not any information about specific variants.
- ADD-IN: This method returns an encrypted and shuffled vector containing zeros for variants that match and randomized non-zero values for those that do not. Decryption reveals the matching variants (as zeros) but does not link them to their original location or reveal the genotypes of non-matching variants.

### 5.4 Security against malicious researcher

The primary security goal of this model is to prevent a researcher from learning information beyond their authorized scope of research. In our defined clinical use case, a researcher has a pre-approved relationship with the family or cohort being studied. Therefore, attacks aimed at re-identifying this specific cohort (e.g. membership inference or uniqueness attacks) are considered out of scope for this initial analysis. The central threat we address is the potential for a researcher to misuse their legitimate access to learn about other patients in the database or to discover incidental findings outside the family’s consent.

Beyond protecting data from outside attackers, the PRISM framework includes a multi-layered security model to prevent misuse by authorized users, such as a curious researcher. Our model assumes a researcher is “honest-but-curious”, meaning they have a legitimate reason to use the system but might try to learn information beyond the scope of their approved research. We defend against this threat using a combination of strong cryptography and a robust governance framework enforced by the Key Management Committee.

#### 5.4.1 Principle of authorized access

A researcher is not given open access to the entire database. Instead, the governance protocol is based on a “need-to-know” principle. Before any analysis can be performed, the researcher must submit a request to study a specific family or cohort, and this request must be approved by the Key Management Committee. This ensures that a researcher’s access is strictly limited to the samples they are authorized to study, preventing them from exploring the data of other patients.

#### 5.4.2 Query review and bundling policy

To prevent a researcher from trying to learn sensitive information about other patients in the database, the committee enforces two important policies:

- Mandatory query review: A researcher must submit their analysis plan to the committee for approval. The plan must justify the scientific reason for the query and define its scope. This review ensures that the analysis is appropriate and not designed to probe for unauthorized information.
- Query bundling: The system is designed for large-scale analysis. Single-variant or single-gene queries are forbidden. Researchers are required to perform analysis on a broad scale (e.g. genome-wide or exome-wide). This “hiding in the crowd” approach makes it infeasible for a researcher to target specific variants to learn about individuals outside of their approved cohort.

#### 5.4.3 Automated filtering of incidental findings

A key feature of our security model is the protection of patients from receiving unsolicited information about their health. The final list of candidate variants is not immediately released to the researcher after decryption. Instead, the process is as follows:

- This list of candidate variants is processed by an automated script that checks the variants against a database of known disease-causing mutations (e.g. ClinVar).
- Variants associated with conditions outside the scope of the original research consent are automatically removed from the list. This respects the patient’s “right not to know” about incidental findings.
- Only the final, sanitized list of relevant or unknown variants is released to the researcher for clinical review.

This combination of controlled access, query oversight, and automated filtering ensures that the privacy of all patients is protected, while still allowing researchers to securely access the critical data they need to make rare disease discoveries.

## 6 Experiments and results

Experiments ran on a Google Cloud high-CPU instance (360 vCPUs, AMD Genoa, 708 GB RAM). We used the BFV-RNS (Bajard *et al.* 2017) scheme via OpenFHE (Badawi et al. 2022). Plaintexts were encrypted at 128-bit security level with multiplicative depth 2 (ADD-IN) or 12 (MUL-IN). OpenMP (https://www.openmp.org/) enabled multithreading (256 threads). Runtimes can be improved with more cores/advanced hardware.

### 6.1 Experiments on real data

We evaluated PRISM on exome data from six cerebrofaciothoracic dysplasia patients (Alanay *et al.* 2014). Variants were annotated using VEP (McLaren *et al.* 2016) (Ensembl 91, hg19). We filtered for rare (freq <5%), high/moderate impact variants using filter_vep and VCF Explorer (Akgün and Demirci 2017). Focusing on the known recessive cause (*TMCO1* mutation (Alanay *et al.* 2014)), our secure recessive operation (MUL-IN method) on 6.4M variant vectors accurately identified the causal gene (see Table 2).

**Table 2.** Results of privacy-preserving recessive operation on real patient data.

Trio	Member	#Rare variants	#Revealed variants	Gene^a^	Ex. time (min)
1	Mother	860	12	N/R	1.831
	Father	967	12	N/R	
	Proband	1053	12	**TMCO1**	
				MUC12	
				UBXN11	
2	Mother	1062	7	N/R	1.827
	Father	906	7	N/R	
	Proband	1035	7	**TMCO1**	
				MUC12	

a Proven causal gene name is highlighted.

Our analysis reveals recessive mutations on TMCO1 gene which are causative mutations of cerebrofaciothoracic dysplasia.

### 6.2 Experiments for recessive and dominant models

**Figure 3.**
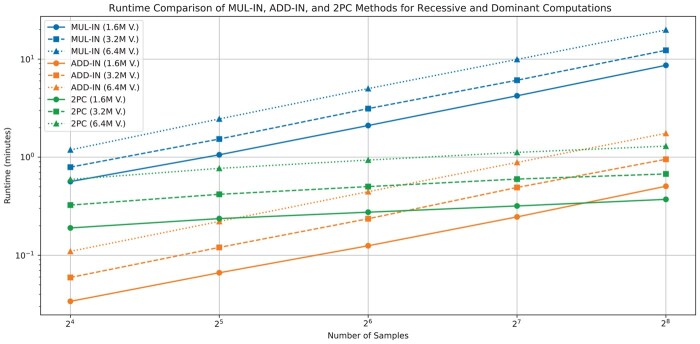
Runtime comparison of the proposed MUL-IN and ADD-IN methods with the 2PC-based solution from (Akgün *et al.* 2021) for rare disease analysis under recessive and dominant inheritance models. Results are presented for three different variant sizes (1.6M, 3.2M, and 6.4M) and sample sizes ranging from 16 to 256.

We compare MUL-IN and ADD-IN performance with the 2PC solution from (Akgün *et al.* 2021), the only prior work on privacy-preserving inheritance model filtering. Runtime trends (server-side computation only) versus variant/sample size are shown (Fig. 3).

The 2PC setup (Akgün *et al.* 2021) used two geographically distinct Google Cloud servers (44 vCPUs, 176GB RAM, 127 ms latency, 154 Mbps bandwidth).

PRISM runtimes increase near-linearly with variants/samples. ADD-IN significantly outperforms MUL-IN. ADD-IN is comparable to 2PC (Akgün *et al.* 2021) up to 128 samples, but 2PC shows better runtime scaling beyond 256 samples (Fig. 3). This reflects a key tradeoff: 2PC’s potentially better computational scaling requires two strictly non-colluding servers and interactive communication (see Section 7), while FHE (PRISM) uses a single-server model without interaction during computation, relying on FHE’s cryptographic security (Section 5). The choice depends on deployment constraints, latency tolerance, and trust assumptions.

### 6.3 Experiments for *de novo* inheritance model

**Figure 4.**
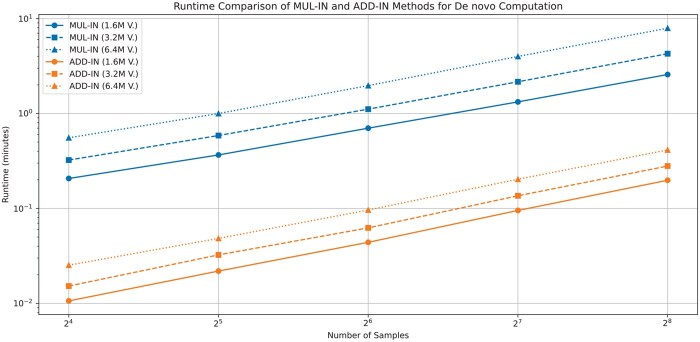
Runtime comparison of the proposed MUL-IN and ADD-IN methods for rare disease analysis under *de novo* inheritance model. Results are presented for three different variant sizes (1.6M, 3.2M, and 6.4M) and sample sizes ranging from 16 to 256.

We evaluate PRISM’s MUL-IN and ADD-IN for *de novo* computation (not supported by (Akgün *et al.* 2021)). Figure 4 shows results. MUL-IN runtime grows linearly; ADD-IN shows much flatter growth, indicating superior efficiency and scalability due to minimized multiplications. ADD-IN significantly outperforms MUL-IN across all tests. Runtimes are practical despite FHE’s computational cost.

## 7 Discussion

Experiments confirm ADD-IN is faster than MUL-IN for all models (up to 17× for recessive/dominant, 22× for *de novo*). For example for 1.6M variants/128 samples (recessive/dominant), ADD-IN took 15 s versus 4 min 14 s for MUL-IN. For 6.4M variants/16 samples (*de novo*), ADD-IN took 1.5 s versus 33 s for MUL-IN.

Homomorphic operation counts (Tables 3 and 4) explain performance differences. Recessive/dominant models require more operations than *de novo*. ADD-IN trades faster computation for larger result ciphertexts and slightly longer decryption times (Tables 5 and 6), which also indicate final download sizes.

**Table 3.** Comparison of the homomorphic operation complexities of the MUL-IN and ADD-IN methods (recessive and dominant inheritance models).

Homomorphic operation	Number of operations (MUL-IN method)	Number of operations (ADD-IN method)
Multiplication	4s−1	2s+1
Addition	6s+v−1	8·s

**Table 4.** Comparison of the homomorphic operation complexities of the MUL-IN and ADD-IN methods (*de novo* inheritance model).

Homomorphic operation	Number of operations (MUL-IN method)	Number of operations (ADD-IN method)
Multiplication	2a+2u−2	2
Addition	2u+v	2a+4u

**Table 5.** Comparison of the resulting ciphertext sizes and interactive decryption times of the MUL-IN and ADD-IN methods (recessive and dominant inheritance models).

Number of variants	Number of committee members	Ciphertext size (MUL-IN)	Dec. time (MUL-IN)	Ciphertext size (ADD-IN)	Dec. time (ADD-IN)
1 600 000	4	10.5 MB	222 ms	201 MB	372 ms
3 200 000	4	10.5 MB	218 ms	403 MB	698 ms
6 400 000	4	10.5 MB	216 ms	805 MB	1 s 258 ms

**Table 6.** Comparison of the resulting ciphertext sizes and interactive decryption times of the MUL-IN and ADD-IN methods (*de novo* inheritance model).

Number of variants	Number of committee members	Ciphertext size (MUL-IN)	Dec. time (MUL-IN)	Ciphertext size (ADD-IN)	Dec. time (ADD-IN)
1 600 000	4	10.5 MB	218 ms	201 MB	359 ms
3 200 000	4	10.5 MB	220 ms	403 MB	665 ms
6 400 000	4	10.5 MB	219 ms	805 MB	1 s 289 ms

The potentially large size of the ciphertext, particularly for the ADD-IN method, may present issues when sharing the encrypted result, increasing download times. However, as seen in Tables 5 and 6, the download sizes (ranging from 10 MB for MUL-IN to 805 MB for ADD-IN with 6.4M variants) may be manageable depending on network infrastructure. Moreover, although the ADD-IN methods increase decryption times compared to MUL-IN, these remain within a reasonable range (e.g. under 1.5 s for 6.4M variants).

Communication patterns also differ between FHE and MPC. PRISM requires a large, one-time encrypted data upload per institution (805 MB for 6.4M variants), then downloads the final result. MPC (Akgün *et al.* 2021) involves multiple interactive communication rounds between servers during computation, sensitive to network latency (127 ms simulated in (Akgün *et al.* 2021)). PRISM trades higher initial transfer for minimal communication/latency dependence during computation.

Using 256 threads, runtimes scale well. With more cores (e.g. 1024), ADD-IN processes 4096 samples/6.4M variants in minutes (7.05 min recessive/dominant, 1.65 min *de novo*). This underscores applicability on accessible high-performance servers for medical applications.

Furthermore, our results demonstrate the practical performance tradeoffs of the Threshold HE model. As shown in Table 7, the decryption time for the ADD-IN method increases from 508 ms with two committee members to 979 ms with eight members. This overhead is a direct result of the interactive, multi-party decryption protocol, where partial decryptions must be generated by and collected from each participating member. This modest increase in time is the price for the significantly enhanced security and decentralized trust that the threshold model provides, successfully moving the system’s security from a policy-based to a cryptographically-enforced guarantee.

**Table 7.** Comparison of the resulting ciphertext, public key, and secret key sizes and interactive decryption times of the MUL-IN and ADD-IN methods (recessive and dominant inheritance models).

Filtering method	Number of variants	Number of committee members	Ciphertext size (MB)	Public key size (MB)	Secret key size (MB)	Decryption time (ms)
MUL-IN	3 200 000	2	10.5	10.5	5.3	214
MUL-IN	3 200 000	4	10.5	10.5	5.3	218
MUL-IN	3 200 000	8	10.5	10.5	5.3	224
ADD-IN	3 200 000	2	403	3.2	1.6	508
ADD-IN	3 200 000	4	403	3.2	1.6	698
ADD-IN	3 200 000	8	403	3.2	1.6	979

## 8 Conclusion

Rare diseases pose significant health risks, and collaborative genomic research is vital but hampered by privacy constraints. We presented PRISM, an FHE-based framework securely identifying disease variants under recessive, dominant, and *de novo* models. Our optimized ADD-IN algorithms offer significant speedups (up to 17–22×) over a multiplication-heavy approach, handling millions of variants efficiently via parallelization despite larger ciphertexts. To our knowledge, this is the first work demonstrating practical FHE feasibility for rare disease analysis across multiple inheritance models. Future work includes extending our methods to more complex inheritance patterns and further performance tuning.

Crucially, PRISM demonstrates how to build such a system with a decentralized trust model using Threshold FHE, moving beyond policy-based trust to provide strong cryptographic guarantees against unauthorized data access. This work highlights the practical feasibility of deploying advanced cryptographic techniques to securely advance rare disease research under strict privacy regulations.

## Data Availability

No new data were generated or analysed in support of this research.
